# Deep Learning based hybrid residual attention and echo state network for high-accuracy heart disease prediction

**DOI:** 10.12688/f1000research.165575.1

**Published:** 2025-07-03

**Authors:** Cenitta D, VIijaya Arjunan Ranganathan, Tanuja Shailesh, Andrew J, Arul N, Praveen Pai T

**Affiliations:** 1 Manipal Institute of Technology, Manipal Academy of Higher Education, Manipal, India; 2 Computer Science and Engineering, AJ Institute of Engineering and Technology, Mangalore, Karnataka, India

**Keywords:** UCI, Kaggle, Heart Disease, Imputation, Deep Learning, Echo State Network, Residual Attention.

## Abstract

**Background:**

Early and accurate prediction of ischemic heart disease (IHD) is essential for reducing mortality and enabling timely intervention. Misdiagnosis can lead to severe health outcomes, emphasizing the need for robust and intelligent predictive models. Deep learning approaches have shown strong potential in identifying hidden patterns in medical data and aiding clinical decision-making.

**Methods:**

This study proposes a novel Hybrid Residual Attention with Echo State Network (HRAESN) model that integrates Attention Residual Learning (ARL) with Echo State Networks (ESN) to enhance feature extraction and temporal data learning. The hybrid model is designed to refine feature attention through residual learning while leveraging ESN for efficient time-series prediction. Two publicly available benchmark datasets were used for evaluation: the Kaggle Cardiovascular Disease dataset comprising 70,000 instances and the UCI Heart Disease dataset containing 303 instances. Missing values in both datasets were handled using a multiple imputation technique tailored for ischemic heart disease. Model performance was assessed using standard classification metrics, including accuracy, sensitivity, specificity, precision, recall, and F-measure.

**Results:**

The proposed HRAESN model demonstrated superior classification performance compared to traditional machine learning and deep learning approaches. It achieved an accuracy of 98.4% on the Kaggle dataset and 97.7% on the UCI dataset. Additionally, the model showed high sensitivity and specificity, indicating strong diagnostic capability and reliability in identifying both diseased and non-diseased cases.

**Conclusions:**

The HRAESN model effectively combines the strengths of residual attention mechanisms and echo state networks, resulting in improved accuracy and stability for ischemic heart disease prediction. Its strong performance on benchmark datasets confirms its potential as a valuable clinical decision support tool for early detection of IHD. Future work may focus on optimizing model complexity and integrating real-time medical IoT data to enhance practical deployment in healthcare systems.

## 1. Introduction

People who develop Ischemic heart disease experience limited blood circulation within specific parts of their body structure. The reduced flow of blood along with diminished oxygen levels to the heart muscle causes cardiac ischemia mostly because of blocked coronary arteries. The persistent reduction of oxygen delivery to the heart through coronary arteries results in coronary artery disease or coronary heart disease leading to heart attack development. Silent ischemia affects numerous individuals who endure heart blood flow interruptions which occur without showing any indicators. Such individuals face risk of experiencing sudden cardiac events easily. The occurrence of silent ischemic events is more frequent in diabetic patients and in individuals who have suffered heart attacks previously. Standard diagnostic techniques consisting of stress tests and Holter monitoring assist medical practitioners in detecting this condition. The Holter monitor represents a portable ECG tool with built-in battery power which tracks heart activity throughout 24 to 48 hours to identify blood flow irregularities. The severity of symptoms determines what diagnostic tests will be used for the evaluation. ^1^

The World Health Organization (WHO) reports that cardiovascular diseases (CVDs) continue as the main cause of global mortality since 17.9 million people died from CVDs in 2019 which amounted to 32% of worldwide fatalities. Heart attacks and strokes lead to 85% of fatal outcomes among the tested patients. ^2^ The worldwide fatalities from noncommunicable diseases reached 17 million during 2019 before people turned 70 years old and cardiovascular conditions caused 38% of those premature deaths. Medical detection of CVDs remains vital because behavioral prevention through risk control methods such as smoking and food control and weight management cannot substitute for early medical discovery to achieve both effective treatment and lower mortality rates. Heart disease poses a major financial challenge and increasing health burden because of high surgical expenses and rising population incidence mainly affecting developing countries. Knowledge about how patient characteristics link to heart disease risk serves as the basis for preventing the condition and detecting it early for treatment purposes.

Deep learning has become an integral part of computer vision, object recognition, natural language processing, speech recognition, medical diagnostics, bioinformatics, and drug discovery. Similar to traditional artificial neural networks (ANNs), deep learning models consist of input, hidden, and output layers, with patient risk factors serving as input features. The research demonstrates that artificial neural networks deliver outstanding results when used for identifying and foretelling coronary heart disease. ^3^ Medical AI applications experience rapid growth because of three main factors including Internet of Things (IoT) and powerful computing hardware (e.g., GPUs and TPUs) together with big medical datasets. Essential information needed by deep learning models comes from Medical IoT devices together with electronic health records as well as genomic data and central medical databases. The critical challenges include preserving data privacy as well as successfully deploying the models and optimizing service quality despite their importance. ^3^

Time-series prediction has seen increased popularity among researchers who use recurrent neural networks (RNNs) as deep learning-based approaches. RNNs work with sequential data sets through the process of feeding output data from previous components to next steps making them ideal for ECG signal processing and patient health surveillance. RNNs differ from regular neural networks by retaining previous input data thus they produce enhanced forecasts for temporal information patterns. Traditional RNNs experience gradient vanishing problems because of which they become problematic for handling long sequences. The development of both Hochreiter and Schmidhuber led to long short-term memory (LSTM) networks which incorporated memory gates to control information transmission and suppress gradient deterioration. ^4^

Time-series extrapolation along with fast learning occurs efficiently through Echo State Networks (ESN) which function as a preferred substitute to normal RNNs. ^5^ An Echo State Network functions through its reservoir of recurrent neurons connected haphazardly that helps the network learn complex patterns yet uses few processing resources. The forecast capabilities of time-series prediction and representation learning capabilities improve through the use of Deep ESNs (DESNs) that include multiple serially connected reservoirs. ^6^

A transformation of conventional convolutional neural networks (CNNs) called Residual Attention Network brings attention mechanism integration for feature enhancement. ^7^ The advanced feed-forward framework permits end-to-end training which enables it to learn hierarchical features independently. Gremlin Deep Residual Attention Networks provide an efficient mechanism for deep learning systems to reach hundreds of layers through their implementation of Attention Residual Learning (ARL). ^8^ Different algorithms can achieve maximum strength performance through hybrid deep learning models which integrate multiple techniques. Medical diagnostic accuracy along with efficiency can experience significant improvement by combining residual attention learning methods with Echo State Networks. The appropriate addressing of missing values through the Ischemic Heart Disease Multiple Imputation Technique creates improved data reliability and completeness. ^9^

### 1.1 Objective of this study

The main goal of this research work is to create a Hybrid Residual Attention with Echo State Network (HRAESN) model used to predict ischemic heart disease (IHD) at an early stage while maintaining high accuracy. The proposed method integrates Residual Attention Learning (RAL) with Echo State Networks (ESNs) to boost both feature extraction and time-series classification and general model performance. This study solves data preprocessing problems with Ischemic Heart Disease Multiple Imputation Technique while using hybrid deep learning effectively for robust classification. The research uses two recognized heart disease data sets including 70,000 records from the Kaggle Cardiovascular Disease dataset and 303 records from the UCI Heart Disease dataset to evaluate the proposed method. The objective is to prove that this approach outperforms current state-of-the-art heart disease prediction methods. ART-based analysis findings will enhance clinical diagnosis along with IHD detection and patient care through AI-powered diagnostic systems.

The following research questions are the focus of the study’s search and synthesis of the literature.

1. How do deep learning models, particularly Echo State Networks (ESNs) and Residual Attention Learning (RAL), improve the accuracy and stability of ischemic heart disease prediction compared to traditional machine learning approaches?
2. What are the key challenges associated with handling missing data in medical datasets, and how can the Ischemic Heart Disease Multiple Imputation Technique enhance data completeness and reliability?
3. How does the proposed Hybrid Residual Attention with Echo State Network (HRAESN) model perform on benchmark datasets (Kaggle Cardiovascular Disease and UCI Heart Disease) compared to existing state-of-the-art heart disease prediction models?

### 1.2 Problem statement

One of the main causes of death is ischemic heart disease (IHD), which needs to be predicted early and accurately in order to be effectively treated. While current machine learning models have trouble managing missing data, time-series dependencies, and computational inefficiencies, traditional diagnostic techniques are costly, time-consuming, and rely on expert interpretation. Vanishing gradients and high complexity are two drawbacks of deep learning techniques like recurrent neural networks (RNNs) and long short-term memory (LSTM) networks. To address these challenges, this study proposes a Hybrid Residual Attention with Echo State Network (HRAESN) model, integrating Residual Attention Learning (RAL) for feature extraction and Echo State Networks (ESNs) for efficient time-series processing, ensuring improved predictive accuracy and robustness.

## 2. Related works

Scientific studies have evaluated multiple deep learning and machine learning prediction methods for heart disease since the turn of the century. The use of recent hybrid deep learning models leads to improved IHD diagnosis accuracy by incorporating various learning techniques. These diagnostic techniques strive to identify the disease at an early stage to enhance medical choices made by healthcare professionals. The research by Li et al. ^10^ presented an S-shaped reconstruction model for arrhythmia detection which employed a 2D 19-layer deep squeeze-and-excitation residual network for predicting heartbeat rates. The authors showed through their work that S-shaped reconstruction demonstrated effective extraction of vital features in ECG heartbeat signals. Self-supervised learning needs additional evolution to complete the enhancement of model classification effectiveness. Their method enables improved feature extraction through graph-based learning which works without needing complete understanding about graph structure in advance. ^11^

Ruobin et al. ^5^ designed a two-stage heart disease forecasting system which combines Empirical Wavelet Transformation (EWT) with Echo State Network (ESN). Empirical Wavelet Transformation-ESN models validated their superiority over traditional forecasting methods during their experimental research. RAGCN serves as the title of Bing et al.’s ^12^ study which developed a classification method for internet services based on Residual Attention Graph Convolutional Network technology featuring attention mechanisms to dynamically weight neighboring nodes yet maintain efficient operation. Sun et al. ^13^ developed the Deep Belief Echo-State Network (DBEN) which utilized Deep Belief Networks (DBN) for feature extraction and Echo State Networks (ESN) for fast learning operations during time series prediction. Short-term memory capacity increased and learning speed and prediction accuracy improved when using DBEN according to their recorded results. The process of achieving optimal parameters for DBEN represents an unresolved issue.

Anguo et al. ^14^ developed a high-precision computing system for time series classification through their work that employed deep CNN models together with reservoir computing and spike encoding features. ^15, 16^ The researchers at Qiang et al. ^17^ introduced a Deep Bidirectional Echo State Network (DBESN) to execute forecasting tasks using scarce data. The proposed system combines Deep Autoencoder Echo State Networks (DAESN) with Deep Bidirectional State Echo State Networks (DBSESN) to find forward and backward time-scale features. Ren et al. implemented a Divided Adaptive Multi-Objective Differential Evolution (DAMODE) classifier that optimizes ESN reservoir constraints resulting in excellent generalizability and classification accuracy according to. ^18, 19^ The researchers at Anusha et al. ^20^ created a deep learning system using DBNs and RNNs for making features and categories. The authors implemented feature weight optimization using Jaya Algorithm-based Multi-Verse Optimization (JA-MVO) technology to surpass previous versions of the model.

The research of Chandrasekaran et al. ^21^ created an on-chip mixed-signal Echo State Network for detecting early cardiac diseases. ^21^ The low-power model of their ESN outperformed deep neural networks (DNNs) in terms of processing efficiency. By integrating Deep Residual Network with Attention Mechanisms Liu et al. ^22^ developed a system for detecting heart diseases in ECG signals through ensemble learning to boost classification results. Chunyan et al. ^23^ designed Recursion-Enhanced Random Forest with an Improved Linear Model (RFRF-ILM) to integrate various feature combinations for an improved heart disease prediction accuracy. Real-time CVD monitoring presents itself as an advantageous application of the Internet of Medical Things according to their research. The research study led by Tamilarasi et al. developed a cardiac disease classification model that unites Random Forest (RF) and Support Vector Machines (SVM) technologies. Their approach for achieving improved forecast accuracy included a feature elimination process that repeated continuously. SVM models are affected by the hyperparameter C and gamma values which leads to instability problems according to their study. ^24^

Girish et al. ^25^ presented a combined deep hybrid model consisting of RNN and LSTM architecture for heart disease diagnosis. Their work employed cross-validation methodologies & data preprocessing strategies to handle intractable data to receive better results of classification rate compared to individual use of ML tools. A hybrid clustering technique that uses; ECG signals and numerical data was proposed by Ritesh et al. ^26^ for predicting cardiac disease. They introduced a new approach which enhanced prediction accuracy through a merged approach of Density-Based Spatial Clustering of Applications with Noise (DBSCAN) and the optimized K-Means Clustering (KMC). Though, their protocol had less specificity than other methods suggest that future studies should look for extra hybrid clustering methods that could be more precise.

Scientific teams have implemented state-of-the-art machine learning algorithms to develop hybrid detection systems that evaluate heart disease through medical data and ECG signals. The predictive accuracy of heart disease diagnosis has been improved by Naïve Bayes (NB) and Random Forest (RF) and Restricted Boltzmann Machines (RBM) techniques according to research findings. ^27, 28^ Decision Tree (DT) classification models have been used to enhance heart disease prediction accuracy according to studies in reference. ^29^ The research of Ritesh et al. ^30^ brought forward a heart disease classification system which merged K-Means as distance-based clustering with DBSCAN as density-based clustering. The hybrid clustering technique achieved better forecasting outcomes than standalone clustering procedures through their implementation.

## 3. Materials and methods

### 3.1 Dataset

This study utilizes data from two publicly available repositories: Kaggle and the UCI (University of California, Irvine) Machine Learning Repository. These datasets provide comprehensive patient records used for cardiovascular disease prediction and ischemic heart disease classification.

**3.1.1 Kaggle cardiovascular disease dataset**

There are 70,000 patient records with 11 distinct features in the Kaggle Cardiovascular Disease dataset. ^31^ When medical practitioners performed clinical examinations, these characteristics were noted. Three types types of input features make up the dataset:

1. Objective Characteristics (Real patient data): Gender, Age, Height, and Weight
2. Features of the Examination (Medical Test Results): Blood Pressure Systolic and Diastolic, Blood Pressure Levels of Cholesterol and Glucose
3. Subjective Features (patient data as self-reported): Alcohol use, smoking, and physical activity

**3.1.2 UCI heart disease dataset**

The UCI Heart Disease dataset contains 76 features, of which 14 are highly relevant for heart disease diagnosis. ^32^ The predictive class attribute is typically listed last, indicating the presence or absence of heart disease. Table 1 and Table 2 provide detailed descriptions of the dataset attributes.

**Table 1.** Kaggle cardiovascular disease dataset description.

Attribute	Description
Age	Objective Feature|age|int (days)
Height	Objective Feature|height|int (cm)|
Weight	Objective Feature|weight|float (kg)|
Gender	Objective Feature|gender|categorical code|
Systolic blood pressure	Examination Feature|ap_hi|int|
Diastolic blood pressure	Examination Feature|ap_lo|int|
Cholesterol	Examination Feature|cholesterol| 1: normal, 2: above normal, 3: well above normal
Glucose	Examination Feature|gluc| 1: normal, 2: above normal, 3: well above normal
Smoking	Subjective Feature|smoke|binary|
Alcohol intake	Subjective Feature|alco|binary|
Physical activity	Subjective Feature|active|binary|
Presence or absence of cardiovascular disease	Target Variable|cardio|binary|

**Table 2.** UCI heart disease dataset description.

Attribute	Description	Domain of value
Age	Age in year	29 to 77
Sex	Sex	Male (1)
		Female (0)
Cp	Chest pain type	Typical angina (1)
		Atypical angina (2)
		Non-anginal (3)
		Asymptomatic (4)
Trestbps	Resting blood sugar	94 to 200 mm Hg
Chol	Serum cholesterol	126 to 564 mg/dl
Fbs	Fasting blood sugar	>120 mg/dl
		True (1)
		False (0)
Restecg	Resting ECG result	Normal (0)
		ST-T wave
		Abnormality (1)
		LV hypertrophy (2)
Thalach	Maximum heart rate achieved	71 to 202
Exang	Exercise induced angina	Yes (1)
		No (0)
Oldpeak	ST depression induced by exercise relative to rest	0 to 6.2
Slope	Slope of peak exercise ST segment	Upsloping (1)
		Flat (2)
		Downsloping (3)
Ca	Number of major vessels coloured by fluoroscopy	0 – 3
Thal	Defect type	Normal (3)
		Fixed defect (6)
		Reversible defect (7)
Num	Heart disease	0-4

**3.1.3 Datasets and ethical considerations**

This study utilizes two publicly available datasets: the Heart Disease dataset from the UCI Machine Learning Repository and the Cardiovascular Disease dataset from Kaggle. These datasets contain anonymized patient records and are publicly released for academic and research purposes.

**3.1.4 Ethical approval statement**

As this research involves only the use of publicly accessible, anonymized datasets, no formal ethical approval was required. The study complies with the ethical principles outlined in the Declaration of Helsinki. No intervention or interaction with human subjects occurred.

**3.1.5 Informed consent statement**

Because this study used pre-existing anonymized data from public repositories, informed consent from participants was not required. All necessary ethical permissions and participant consents were obtained by the original data providers as per their respective institutional and data-sharing policies.

### 3.2 Hybrid data classification algorithm

The classification of ischemic heart disease (IHD) in this study is based on a hybrid deep learning model that integrates machine learning (ML), soft computing techniques, and optimization methods to enhance accuracy and robustness. Different classification models are created by integrating various ML methods and ensemble learning methods that involve bagging and boosting. Multiple classifiers work together in ensemble methods to generate better generalization as well as decrease overfitting.

HRAESN model combines the following key elements:

1. Echo State Networks (ESNs) for efficient time-series processing
2. Attention Residual Learning (ARL) for enhanced feature extraction

By combining ESN and ARL, the model achieves higher accuracy, better generalization, and improved stability compared to conventional ML classifiers.

### 3.3 Echo State Network (ESN)

Echo State Networks (ESNs), a subset of recurrent neural networks (RNNs) created for effective sequential data processing, are a part of the reservoir computing paradigm. In contrast to conventional RNNs, an ESN’s hidden layer (reservoir) is fixed and randomly initialized, whereas only the output layer is trained.

Key features of ESNs include:

• The reservoir exhibits two weight sets which are fixed by random values without training: W_in for input-to-lateral connections and W_r for lateral connections.
• During ESN operation researchers only train output weights but maintain simple computational design for efficient pattern learning capability.
• The hidden layer connectivity of ESNs remains sparse which decreases computational complexity.
• Nonlinear Embedding: The reservoir state provides a nonlinear transformation of input data, which can then be mapped to the desired output using a trainable readout layer.
  Since ESNs retain past information in a fixed reservoir, they are highly effective for time-series forecasting and real-time signal processing, making them a suitable choice for ischemic heart disease prediction.

### 3.4 Attention Residual Learning (ARL)

Attention Residual Learning (ARL) is a deep learning technique that enhances feature extraction by selectively focusing on relevant information while reducing noise in deep neural networks. It is particularly beneficial in medical image analysis and time-series classification.

Key challenges in deep residual networks include:

• Performance Degradation: Stacking multiple narrow attention modules can lead to a decline in performance.
• Feature Suppression: Soft mask layers may inadvertently reduce the importance of relevant features.

To address these issues, ARL modifies feature representation using an attention mask. The transformation is mathematically represented as:

$$H_{i}\left(t+1\right)=\left(1+M_{i}\left(t\right)\right)*X_{F}^{i}\left(t\right)$$

Where:

i: Index position in the input matrix

M _i_ (t): Gradient of the input feature mask during the t-th iteration

H _i_ (t+1): Updated attention module output at the (t+1)-th iteration

This formulation ensures that:

1. Relevant features are amplified, while irrelevant features are suppressed.
2. Deep residual networks maintain stable performance even with hundreds of layers.
3. Computational efficiency is preserved without significantly increasing model complexity.

The integration of ESNs with ARL enables the proposed HRAESN model to merge its time-series learning functionality with attention-based feature refinement that results in precise and stable outcomes for ischemic heart disease predictions.

### 3.5 Methodology

The prediction model utilizes heart disease records from UCI Heart Disease Data Set and the Cardiovascular Disease dataset from Kaggle. Pre-processing starts with performing the Ischemic Heart Disease Multiple Imputation Technique to identify and imputation missing values before proceeding further. ^1^ The HRAESN model combines Echo State Networks (ESNs) for short-term memory processing with Attention Residual Learning (ARL) for enhancing features to classify heart disease.

The model’s accuracy, sensitivity, and specificity are assessed using a confusion matrix. The experiment’s process is shown in Figure 1, and the HRAESN classifier’s overall system model is shown in Figure 2.

**Figure 1.**
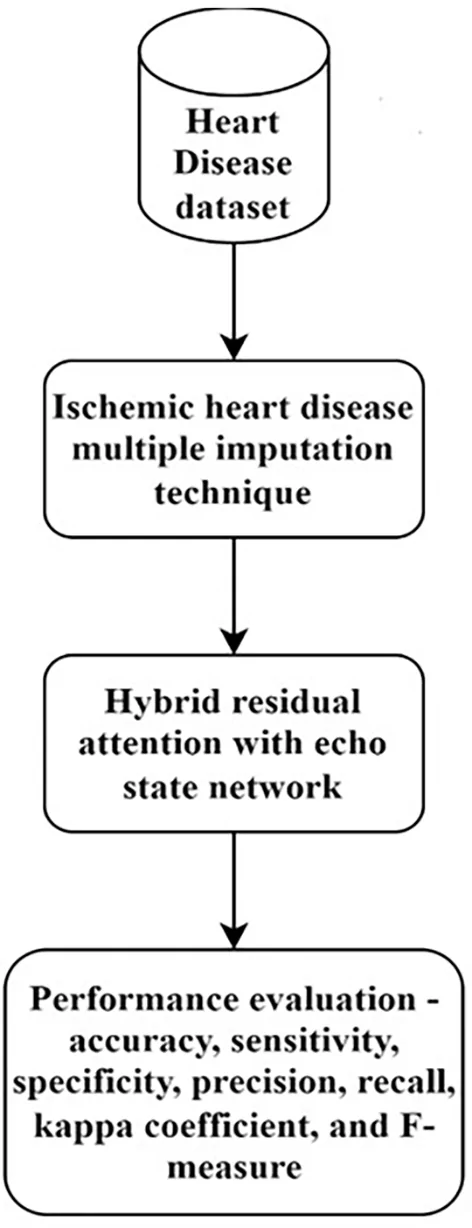
Workflow of the proposed experiment using the UCI heart disease and Kaggle cardiovascular disease datasets.

**Figure 2.**
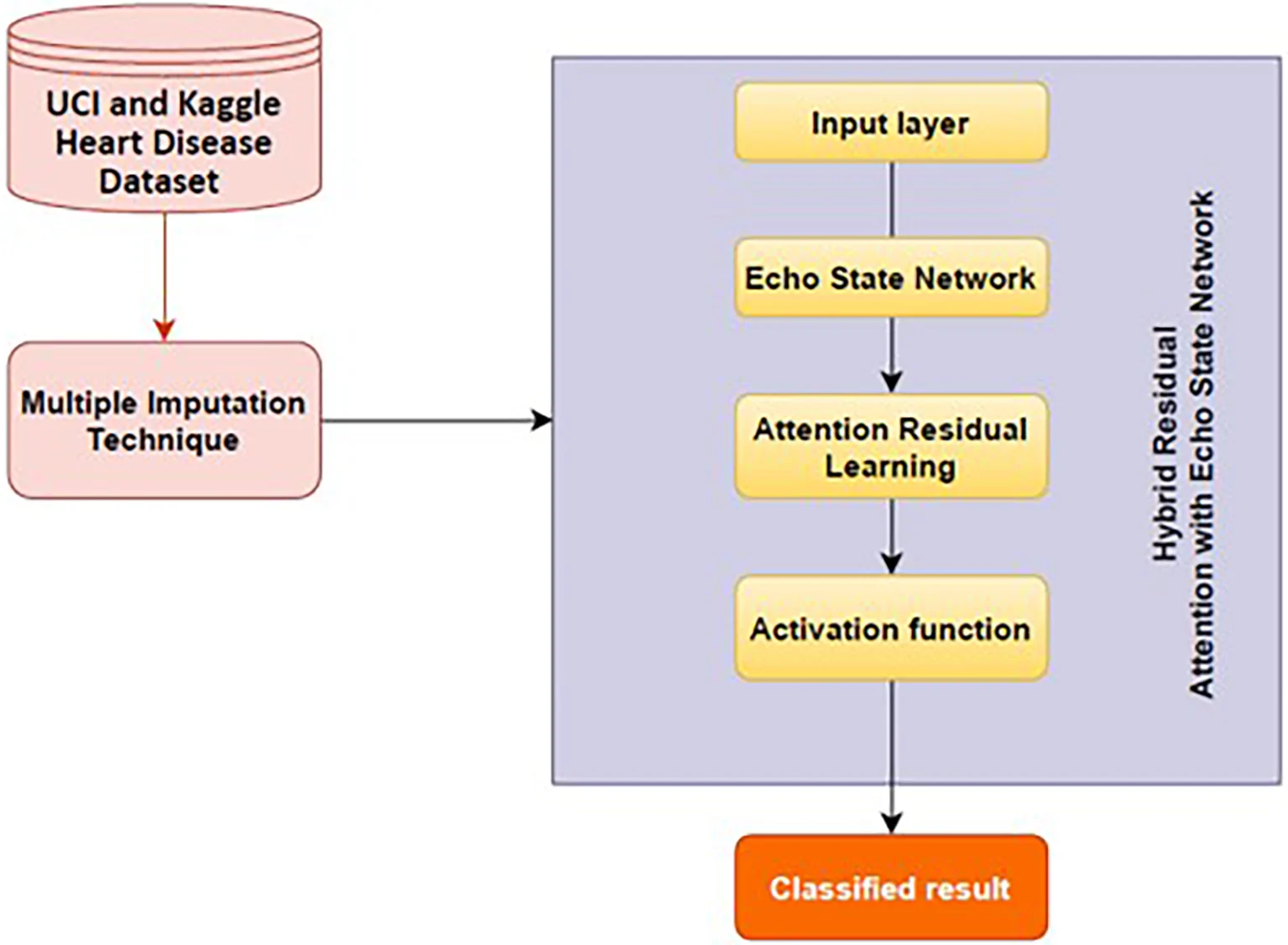
Overall system model of the Hybrid Residual Attention with Echo State Network (HRAESN).

*Experiment workflow*

1. Load and preprocess datasets: The Heart Disease Data Set and Cardiovascular Disease dataset are loaded, and missing values are imputed using the Ischemic Heart Disease Multiple Imputation Technique. ^9, 33^
2. Feature extraction and classification: The HRAESN model applies ESNs for sequence modeling and ARL for refining feature representation.
3. Model evaluation: A confusion matrix assesses the model’s performance, ensuring accurate classification of heart disease cases.

**3.5.1 Hybrid Residual Attention with Echo State Network (HRAESN) algorithm**

The input feature matrix (X _F_) is obtained from the Ischemic Heart Disease Multiple Imputation Technique and labeled according to class 0 (normal) or class 1 (heart disease).

**Echo State Network (ESN) Hidden Layer Dynamics**

$$X_{F}\left(t+1\right)=f_{a}\left(W^{i}u\left(t\right)+W^{r}X_{F}\left(t\right)\right)$$ (1)

Where:

•
$X_{F}\left(t+1\right)$ and $X_{F}\left(t\right)$ are the feature matrices at iterations t and t + 1.
•
$W^{i}$ is the input reservoir weight matrix derived from the input data.
•
$W^{r}$ is the reservoir weight matrix representing internal states.
•
u(t+1) represents the internal states computed at iteration t.
•
$f_{a}\left(.\right)$ is the activation function applied at the reservoir.

**Attention Residual Learning (ARL) transformation**

$$H_{i}\left(t+1\right)=\left(1+M_{i}\left(t\right)\right)*X_{F}^{i}\left(t\right)$$ (2)

Where:

•
i represents the input matrix’s index positions.
•
$M_{i}\left(t\right)$ is the gradient of the input feature mask at iteration t.
•
$H_{i}\left(t+1\right)$ is the attention module output at iteration t + 1.

The reservoirs in HRAESN are linked in series, meaning each reservoir state depends on the previous reservoir’s output and its own past state:

$$X_{F}^{1}\left(t+1\right)=f_{a}\left(W^{i}u\left(t\right)+W^{1}X_{F}^{1}\left(t\right)\right)$$ (3)

$$X_{F}^{2}\left(t+1\right)=f_{a}\left(W^{i}X_{F}^{1}\left(t\right)+W^{2}X_{F}^{2}\left(t\right)\right)$$ (4)

$$X_{F}^{M}\left(t+1\right)=f_{a}\left(W^{i}X_{F}^{\left(M-1\right)}\left(t\right)+W^{M}X_{F}^{M}\left(t\right)\right)$$ (5)

Where:

•
$W^{i}=H_{i}\left(t+1\right)$ represents the attention module output.

Activation Functions and Output Computation

1. Final Activation Function

$$A_{n}=Y_{L}\cdot \text{sigmoid}\left(X_{F}^{M}\left(t+1\right)\right)$$ (6)

Where:

•
sigmoid(.) is the activation function applied to the final output layer.

Dynamic Echo State Network Output

$$P_{R}\left(t+1\right)=g_{a}\left(W^{o}X_{F}^{M}\left(t+1\right)\right)$$ (7)

Where:

•
$W^{o}$ represents the output reservoir weight matrix.
•
$g_{a}\left(.\right)$ is the final activation function used at step 4.

Algorithm. **Hybrid Residual Attention with Echo State Network (HRAESN).**

**Input:** features data $X_{F}$ , label data $Y_{L}$

**Output:** Predicted result P _r_

1: **begin**

2: **for each** Compute the Hidden layer of dynamic ESN

3: $X_{F}\left(t+1\right)=f_{a}\left(W^{i}u\left(t\right)+W^{r}X_{F}\left(t\right)\right)$

4: **end for**

5: **for each** compute the attention residual learning

6: $H_{i}\left(t+1\right)=\left(1+M_{i}\left(t\right)\right)*X_{F}^{i}\left(t\right)$

7: **end for**

8: **for** x=1 to M **do:**

9: $X_{F}^{1}\left(t+1\right)=f_{a}\left(W^{i}u\left(t\right)+W^{1}X_{F}^{1}\left(t\right)\right)$

10: $X_{F}^{2}\left(t+1\right)=f_{a}\left(W^{i}X_{F}^{1}\left(t\right)+W^{2}X_{F}^{2}\left(t\right)\right)$

11:  …

12: $X_{F}^{M}\left(t+1\right)=f_{a}\left(W^{i}X_{F}^{M-1}\left(t\right)+W^{M}X_{F}^{M}\left(t\right)\right)$

13: **end**

14: **end**

**3.5.2 Hyperparameter tuning**

The Hyperparameter Tuning process optimizes the performance of the Hybrid Residual Attention with Echo State Network (HRAESN) model by carefully selecting key parameters for both Echo State Networks (ESN) and Attention Residual Learning (ARL). The reservoir size (500 neurons) and spectral radius (0.8) ensure stable memory retention for time-series processing, while 10% sparse connectivity enhances computational efficiency. The input scaling (0.5) and leaky rate (0.2) regulate data flow within the reservoir, preventing overfitting. The attention module depth (3 layers) and mask range ([0,1]) refine feature selection, improving model interpretability. The model is trained using the Adam optimizer with a learning rate of 0.001, a batch size of 32, and 100 epochs for optimal convergence. The model prevents overfitting through dropout rate 0.3 while 80:20 train-test split maintains evaluation stability. The optimized parameters lead to precise and efficient and stable ischemic heart disease predictions as described in Table 3.

**Table 3.** Summary of hyperparameter settings used in the proposed model training.

Parameter	Value	Description
**Number of Reservoir Neurons (N_res)**	500	Number of neurons in the ESN reservoir. Determines the capacity of the reservoir to store and process sequential information.
**Spectral Radius (ρ)**	0.8	Controls the stability of the ESN. A value < 1 ensures echo state property for long-term memory.
**Reservoir Connectivity (%)**	10%	Percentage of nonzero connections in the reservoir matrix W ^r^, ensuring sparse connectivity.
**Input Scaling (W_in)**	0.5	Determines how input data is mapped into the reservoir.
**Leaky Rate (α)**	0.2	Defines how much of the previous state is retained in the ESN for time-series processing.
**Readout Regularization (λ)**	10 ^−4^	Ridge regression parameter to prevent overfitting in the output layer of ESN.
**Attention Module Depth**	3	Number of stacked attention modules in ARL to enhance feature learning.
**Attention Mask Range (M_i (t))**	[0,1]	Defines the range of soft masks applied in attention residual learning.
**Activation Function (f_a(.))**	Tanh	Non-linear activation function used in the ESN reservoir.
**Output Activation Function (g_a(.))**	Sigmoid	Activation function used in the final output layer to predict class labels.
**Batch Size**	32	Number of training samples processed before updating model weights.
**Optimizer**	Adam	Optimization algorithm used to update model parameters.
**Learning Rate (η)**	0.001	Controls the step size of weight updates during training.
**Dropout Rate**	0.3	Fraction of neurons randomly dropped during training to prevent overfitting.
**Number of Epochs**	100	Total number of times the model iterates over the entire dataset during training.
**Train-Test Split Ratio**	80:20:00	Data split for training (80%) and testing (20%).

## 4. Results and analysis

To predict the existence of ischemic heart disease (IHD), a number of classification methods were employed, including Naïve Bayes (NB), Support Vector Machine (SVM), Logistic Regression (LR), Random Forest (RF), and AdaBoost. Data from the Cardiovascular Disease dataset (Kaggle) and the Heart Disease Data Set (UCI) were used in the experiments.

### 4.1 Experiment setup and data preprocessing

The datasets contain various medical indicators that serve as input features for classification. The target variable is binary:

• Class 1: Presence of ischemic heart disease
• Class 0: Absence of disease

The proposed hybrid HRAESN model is trained using 80% of the dataset, and the remaining 20% is used for testing. Principal Component Analysis (PCA) was applied to highlight variance and distinct patterns in the dataset. Figure 3 shows the PCA plot, where:

• Principal Component 1 (X-axis) and Principal Component 2 (Y-axis) capture most of the variance.
• Blue (0) represents healthy individuals, while Red (1) represents patients with heart disease.

**Figure 3.**
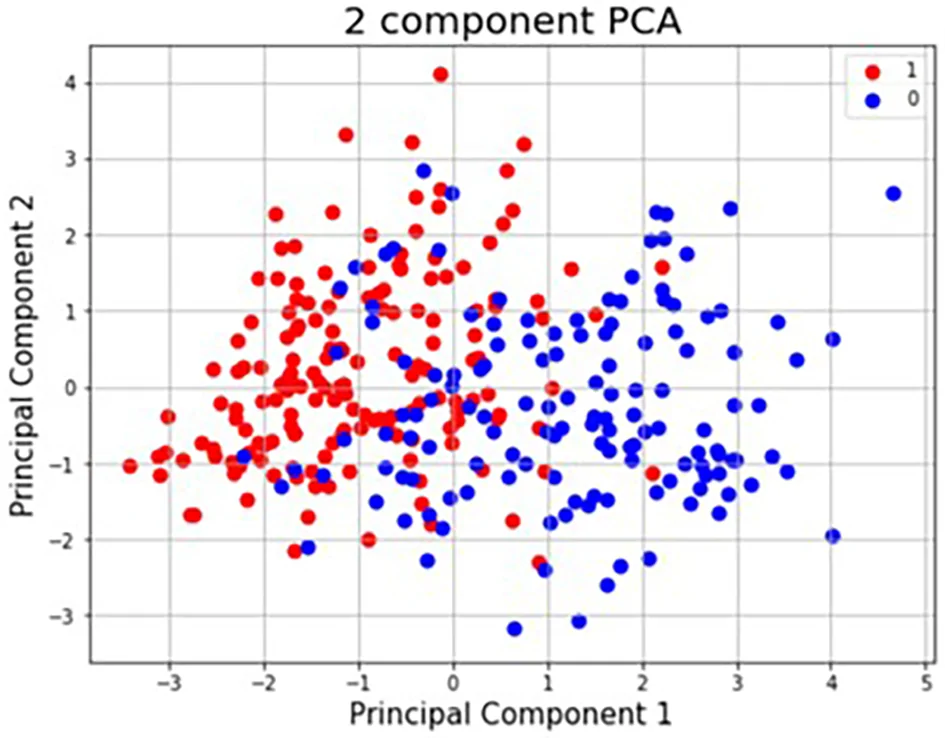
PCA plot showing data distribution in the heart disease dataset based on the first two principal components.

Additionally, six records in the UCI dataset had missing values, which were imputed using the Ischemic Heart Disease Multiple Imputation technique, producing a complete dataset with no missing values.

### 4.2 Experimental results

Tables 4 and 5 present the normalized confusion matrix for the HRAESN model using the UCI Heart Disease dataset and Kaggle Cardiovascular Disease dataset, respectively.

**Table 4.** Normalized confusion matrix for the Hybrid Residual Attention with Echo State Network (HRAESN) using the UCI heart disease datasets.

		Predicted	
		Label	
	Class	0	1
Actual label	0	163	1
	1	3	136

**Table 5.** Normalized confusion matrix for the hybrid residual attention with Echo state network using Kaggle cardiovascular disease dataset.

		Predicted	
		Label	
	Class	0	1
Actual label	0	34431	549
	1	568	34452

Figures 4– 6 illustrate the comparative performance of different classifiers used for ischemic heart disease prediction. Figure 4 evaluates Sensitivity, Specificity, Precision, F-measure, and Accuracy, providing insight into the model’s ability to correctly classify positive and negative cases. Higher values indicate improved diagnostic reliability. ^34^ Figure 5 presents the Kappa coefficient, Recall, and Jaccard coefficient, which measure classifier agreement beyond chance, model recall capability, and overall similarity between predicted and actual values. A higher Kappa score signifies better classifier consistency.

**Figure 4.**
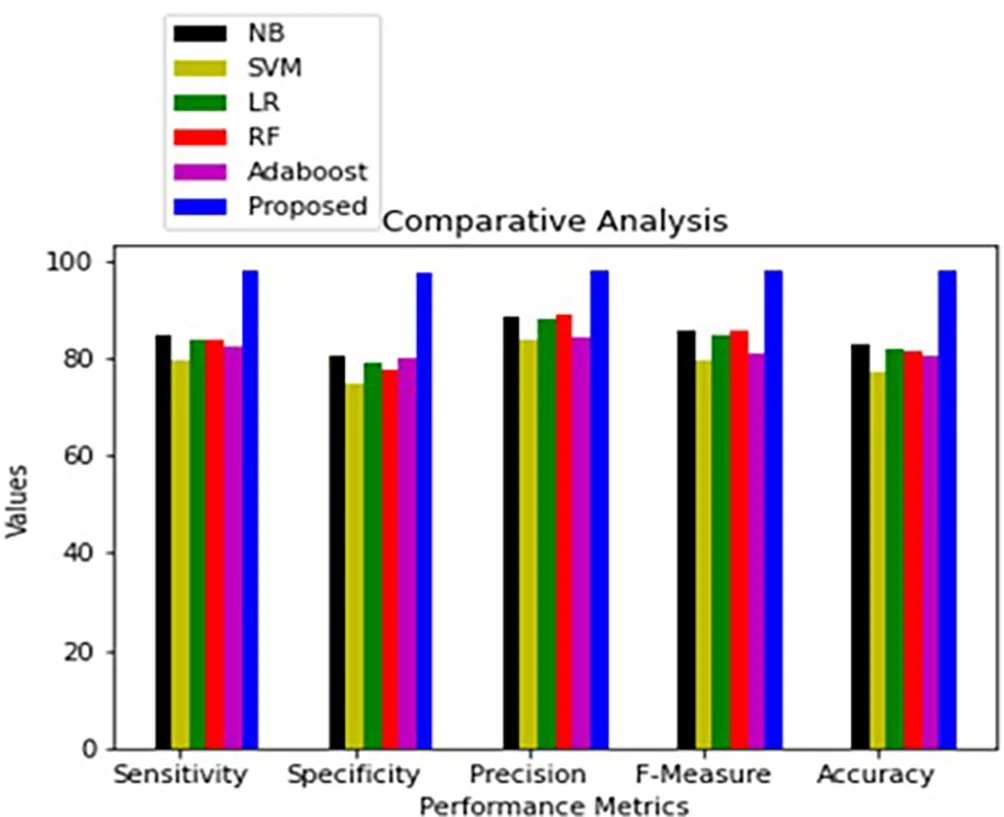
Analysis of classifier performance based on sensitivity, specificity, precision, F-measure, and accuracy.

**Figure 5.**
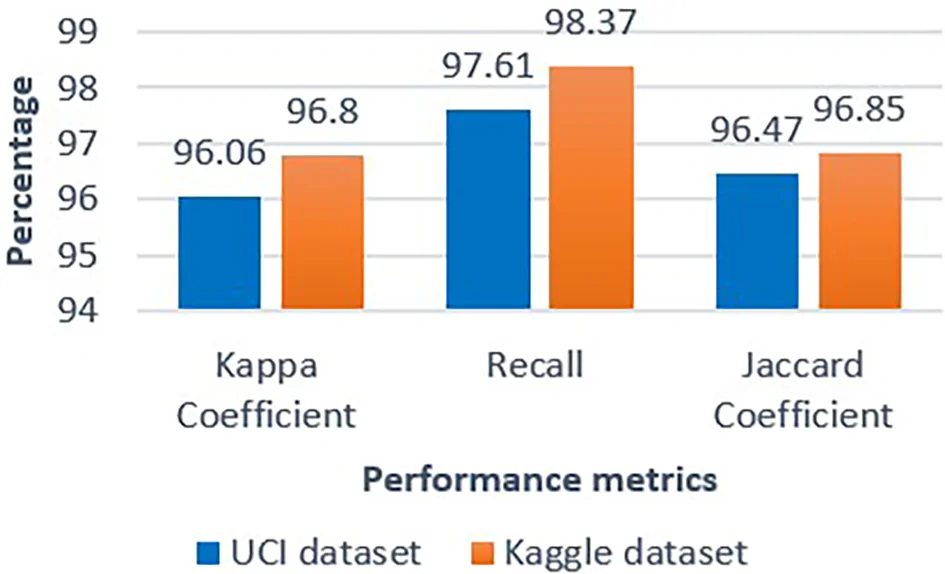
Analysis of classifier performance using Kappa coefficient, Recall, and Jaccard coefficient on UCI heart disease and Kaggle cardiovascular disease datasets.

Figure 6 examines Classification Error Rate, False Acceptance Rate (FAR), and False Rejection Rate (FRR), assessing the model’s robustness against false classifications. A lower FAR and FRR indicate reduced misclassification, ensuring better clinical applicability. These evaluations confirm that the proposed HRAESN model outperforms traditional classifiers in multiple performance aspects.

**Figure 6.**
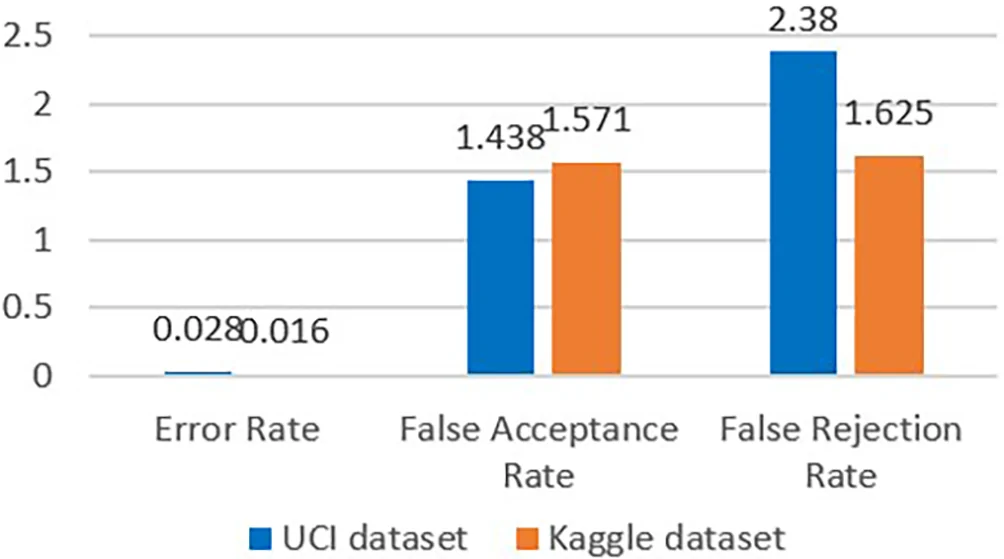
Classification error rate, false acceptance rate, and false rejection rate for UCI heart disease and Kaggle cardiovascular disease datasets.

### 4.3 Comparative analysis with existing models

Tables 6 and 7 present a comparative analysis between the proposed Hybrid Residual Attention with Echo State Network (HRAESN) model and existing heart disease prediction models. The comparison is based on handling of missing values, classifier types, and accuracy performance across different studies. Unlike traditional models that either delete missing data or use basic imputation techniques, the HRAESN model applies a multiple imputation approach, ensuring data completeness and improving prediction reliability. The results indicate that the HRAESN model outperforms previous approaches, achieving 97.71% accuracy on the UCI Heart Disease dataset and 98.4% accuracy on the Kaggle Cardiovascular Disease dataset. Compared to Random Forest (RF), Gradient Boosting (GB), Multilayer Perceptron (MLP), and other ensemble methods, the HRAESN model exhibits superior classification performance, demonstrating its effectiveness in early ischemic heart disease detection and clinical decision support.

**Table 6.** Comparison of HRAESN with existing methods using the UCI heart disease dataset.

Study	Year	Handling of missing values	Classifiers	Accuracy (%)
Jabbar et al. ^35^	2016	Rows with missing values deleted	RF	83.6
Verma & Mathur ^36^	2019	Rows with missing values deleted	MLP	85.48
Latha & Jeeva ^37^	2019	Rows with missing values deleted	Hybrid NB, BN, MLP, RF	85.48
Tama et al. ^38^	2020	Rows with missing values deleted	Two-tier ensemble (RF, GB, XGBoost)	85.71
Pooja et al. ^34^	2021	MICE Algorithm	RF	86.6
Proposed HRAESN	2023	Multiple Imputation Technique	HRAESN	97.71

**Table 7.** Comparison of HRAESN with existing methods using the Kaggle cardiovascular disease dataset.

Study	Year	Classifiers	Accuracy (%)
Maiga et al. ^39^	2019	RF	73
Hagan ^40^	2021	RF, Gradient Boosting	74
Bhoyar ^41^	2021	MLP	89.7
Theerthagiri ^42^	2022	Gradient Boosting	89.7
Mohammed et al. ^43^	2021	Hybrid RF, NB, GB	94
Proposed HRAESN	2023	HRAESN	98.4

**Table 8.** Performance comparison of different algorithms.

Classifiers	Accuracy	Specificity	Sensitivity
**Logistic regression**	83.3	82.3	86.3
**K neighbors**	84.8	77.7	85
**SVM**	83.2	78.7	78.2
**RF**	80.3	78.7	78.2
**DT**	82.3	78.9	78.5
**Deep Learning**	94.2	83.1	82.3
**Proposed HRAESN with UCI dataset**	**97.71**	**98.03**	**97.4**
**Proposed HRAESN with Kaggle dataset**	**98.4**	**98.42**	**98.37**

Figure 7 compares the HRAESN model with Residual Networks (ResNet) and Echo State Networks (ESN) in terms of classification performance. The HRAESN model achieves 0.98, significantly outperforming ESN (0.89) and ResNet (0.75). This improvement demonstrates the effectiveness of combining Echo State Networks with Attention Residual Learning, enhancing feature extraction and time-series prediction. The results confirm that HRAESN provides superior accuracy and stability in ischemic heart disease classification.

**Figure 7.**
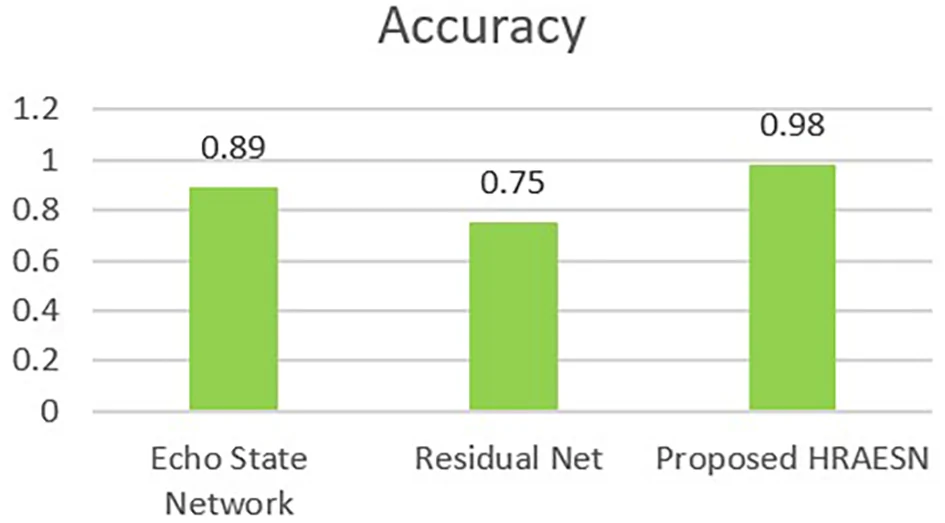
Comparison of residual network, echo state network, and the proposed Hybrid Residual Attention Echo State Network.

## 5. Discussion

The proposed HRAESN model significantly outperforms conventional machine learning and deep learning techniques in ischemic heart disease classification. It achieves higher accuracy, sensitivity, and specificity, as demonstrated in Tables 6– 9. The proposed model exhibits:

• Improved classification accuracy (97.71% – UCI dataset, 98.4% – Kaggle dataset)
• Effective handling of missing data using Multiple Imputation Technique
• Enhanced feature learning through Attention Residual Learning (ARL)
• Better time-series processing with Echo State Networks (ESN)

**Table 9.** Performance of deep learning classifiers on the heart disease dataset.

DL classifiers	Accuracy (%)
Multi-layer perceptron	72.52
Deep neural network (200 epochs)	80.21
Recurrent neural network	88.52
Long sort term memory network	86.88
Hybrid deep learning model (RNN + LSTM)	95.1
Proposed HRAESN	97.71

However, the model has higher computational complexity, which can be optimized in future work. Integrating IoT-based medical devices for real-time heart disease monitoring can further enhance its applicability in healthcare solutions.

## 6. Conclusion

Using the UCI Heart Disease dataset and the Kaggle Cardiovascular Disease dataset, the suggested Hybrid Residual Attention with Echo State Network (HRAESN) model has been compared to several Machine Learning (ML) and Deep Learning (DL) techniques for the classification of Ischemic Heart Disease (IHD). The experimental results demonstrate that HRAESN outpaces existing heart illness prediction methods because it achieves accuracy rates of 98.4% on Kaggle data and 97.7% on UCI data. The HRAESN model demonstrates superior performance in terms of sensitivity together with specificity and recall along with accuracy and F-measure according to deep learning model comparisons. The Ischemic Heart Disease Multiple Imputation Technique incorporated within the model succeeds in handling missing values to achieve better data completeness along with improved predictive reliability.

The HRAESN model demonstrated better testing stability characteristics than conventional classifiers thus establishing itself as a dependable instrument for medical diagnosis and clinical decisions. The model achieves powerful medical dataset pattern detection through the combination of Echo State Networks (ESN) and Attention Residual Learning (ARL) features. The future research should work on optimizing the computational operations and integrating IoT-based medical equipment to detect ischemic heart disease in real-time. This approach demonstrates significant value for healthcare improvements by providing early medical diagnosis together with decreased chances of life-threatening cardiac events.

## Ethical statement

This study did not involve human or animal subjects, and thus no ethical approval was required.

## CRediT authorship contribution statement

D. Cenitta: Methodology and Project administration. R. Vijaya Arjunan: Conceptualization, Writing – review & editing. Tanuja Shailesh: Writing – review & editing. Andrew J: Data curation. N. Arul: Visualization. Praveen Pai T: Review & editing.

## Declaration of competing interest

The authors declare that they have no known competing financial interests or personal relationships that could have appeared to influence the work reported in this paper.

## Disclaimer/publisher’s note

The statements, opinions and data contained in all publications are solely those of the individual author(s) and contributor(s).

## Data Availability

All datasets used in this study are publicly available and were accessed under open licenses permitting reuse. The Heart Disease dataset was obtained from the UCI Machine Learning Repository and can be accessed at: https://archive.ics.uci.edu/ml/datasets/Heart+Disease **Persistent Identifier**: UCI Heart Disease Dataset – DOI: *Not applicable (repository does not assign DOI)* The Cardiovascular Disease dataset was obtained from Kaggle and can be accessed at: https://www.kaggle.com/datasets/sulianova/cardiovascular-disease-dataset **Persistent Identifier**: Kaggle Dataset – DOI: *Not applicable (repository does not assign DOI)* All data supporting the results, including the values used to compute performance metrics (accuracy, sensitivity, specificity, F-measure), build figures (e.g., PCA plots, confusion matrices), and generate tables, are available in the original datasets and fully included in the supplementary materials submitted with this article. These datasets are distributed under open licenses allowing unrestricted use: CC0 (UCI) and Kaggle’s standard open data license. No additional ethical, privacy, or security concerns apply. Both datasets are openly accessible for academic and research purposes and do not contain any personally identifiable information. However, **as the current study is based on third-party data, the authors were not involved in the original data collection process**. To the best of our knowledge:
• The **UCI Heart Disease dataset** was originally contributed by researchers from the **Cleveland Clinic Foundation** and is widely used in medical data mining research. Specific details regarding ethical approval and informed consent for this dataset were not provided in the original UCI repository documentation. • The **Kaggle Cardiovascular Disease dataset** was uploaded by the contributor Y. Suliana, who stated that the data was anonymized and collected during routine clinical practice. However, no specific name of the ethics committee, approval date, or consent procedure is disclosed in the dataset description. As per the policies of UCI and Kaggle, datasets are made publicly available under the assumption that all ethical requirements and informed consent procedures were handled appropriately by the original data custodians. Since no personally identifiable data is included, and the data is anonymized, **no additional ethical approval or consent was required** for our use of these datasets in accordance with our institutional guidelines and the Declaration of Helsinki.
