# Supplementary material for: Deep Learning Based Hybrid Residual Attention and Echo State Network for High-Accuracy Heart Disease Prediction
Source: F1000Res. 2026 Oct 28;14:650. Originally published 2025 Jul 3. [Version 3] doi: 10.12688/f1000research.165575.3 (PMC13691351; doi:10.12688/f1000research.165575.3)
Supplement: Peer review history

# Reviewer report: Reviewer response for version 2

**DOI:** 10.5256/f1000research.187669.r516894

**Published:** 2026-10-22

**Authors:** D. Vijendra Babu

**Affiliations:** 1 Vellore Institute of Technology, Vellore, Tamil Nadu, India

Review Report – Recommendation for Acceptance

 Article Title: Deep Learning Based Hybrid Residual Attention and Echo State Network for High-Accuracy Heart Disease Prediction

1. The manuscript addresses an important and relevant research problem in healthcare through the application of deep learning for early heart disease prediction.
2. The proposed Hybrid Residual Attention with Echo State Network (HRAESN) model presents an innovative integration of attention-based residual learning and echo state networks.
3. The study employs two publicly available benchmark datasets, namely the Kaggle Cardiovascular Disease dataset and the UCI Heart Disease dataset, supporting comparative evaluation.
4. The methodology incorporates missing-data imputation and multiple classification performance metrics, demonstrating a systematic experimental approach.
5. The manuscript provides a comprehensive comparison of the proposed model with conventional machine learning and deep learning techniques.
6. The reported accuracy of 98.4% on the Kaggle dataset and 97.71% on the UCI dataset indicates promising predictive performance.
7. The presentation of confusion matrices, performance comparisons, and evaluation metrics strengthens the empirical analysis.
8. The revised manuscript demonstrates improved methodological transparency, statistical evaluation, and discussion of limitations.
9. The manuscript is logically organized, technically relevant, and contributes meaningfully to research on AI-assisted healthcare decision support.
10. Overall, the article is suitable for indexing, and I recommend its acceptance for indexing as a valuable contribution to deep learning-based heart disease prediction research.

Is the work clearly and accurately presented and does it cite the current literature?

Yes

If applicable, is the statistical analysis and its interpretation appropriate?

Yes

Are all the source data underlying the results available to ensure full reproducibility?

Yes

Is the study design appropriate and is the work technically sound?

Yes

Are the conclusions drawn adequately supported by the results?

Yes

Are sufficient details of methods and analysis provided to allow replication by others?

Yes

Reviewer Expertise:

AI/ML, IoT, Digital Twin, 5G Communication

I confirm that I have read this submission and believe that I have an appropriate level of expertise to confirm that it is of an acceptable scientific standard.

# Response

**Published:** 2026-10-22

**Authors:** VIJAYA ARJUNAN RANGANATHAN

**Affiliations:** School of Computer Engineering, Manipal Academy of Higher Education, Manipal, Karnataka, India

**Deep Learning based hybrid residual attention and echo state network for high-accuracy heart disease prediction**

**Responses to peer review reports**

**Reviewer Concern # 1:** **The manuscript addresses an important and relevant research problem in healthcare through the application of deep learning for early heart disease prediction.**

**Author response:**  We thank the reviewer for recognizing the importance and relevance of the research problem addressed in this study. Our work aims to improve ischemic heart disease prediction through the proposed HRAESN framework.

****

**Reviewer Concern # 2:** **The proposed Hybrid Residual Attention with Echo State Network (HRAESN) model presents an innovative integration of attention-based residual learning and echo state networks.**

**Author response:**  We appreciate the reviewer’s positive assessment of the proposed HRAESN framework. The model integrates Attention Residual Learning for feature extraction with Echo State Networks for learning patterns, with the objective of improving IHD classification performance.

****

**Reviewer Concern # 3:** **The study employs two publicly available benchmark datasets, namely the Kaggle Cardiovascular Disease dataset and the UCI Heart Disease dataset, supporting comparative evaluation.**

**Author response:**  We thank the reviewer for acknowledging the use of two publicly available benchmark datasets. Evaluating the proposed model on both datasets provides an opportunity to assess its performance across different dataset sizes and characteristics.

****

**Reviewer Concern # 4:** **The methodology incorporates missing-data imputation and multiple classification performance metrics, demonstrating a systematic experimental approach.**

**Author response:**  We appreciate the reviewer’s recognition of the missing-data handling procedure and the use of multiple classification metrics. The manuscript describes the imputation technique and evaluates model performance using accuracy, sensitivity, specificity, precision, recall, and F-measure.

****

**Reviewer Concern # 5:** **The manuscript provides a comprehensive comparison of the proposed model with conventional machine learning and deep learning techniques.**

**Author response:**  We thank the reviewer for acknowledging the comparative evaluation of the proposed HRAESN model against existing machine-learning and deep-learning approaches. This evaluation helps demonstrate the model’s predictive performance relative to the methods included in the study.

****

**Reviewer Concern # 6:** **The reported accuracy of 98.4% on the Kaggle dataset and 97.71% on the UCI dataset indicates promising predictive performance.**

**Author response:**  We thank the reviewer for highlighting the predictive performance achieved by the proposed HRAESN model on both benchmark datasets. The reported results demonstrate the model’s performance under the experimental settings described in the manuscript.

**Author action: **Updated Sections 5 and 7.

****

**Reviewer Concern # 7:** **The presentation of confusion matrices, performance comparisons, and evaluation metrics strengthens the empirical analysis.**

**Author response:**  We appreciate the reviewer’s positive assessment of the experimental evaluation. The confusion matrices and classification metrics are included to provide a more detailed assessment of the model’s classification performance.

****

**Reviewer Concern # 8:** **The revised manuscript demonstrates improved methodological transparency, statistical evaluation, and discussion of limitations.**

**Author response:**  We thank the reviewer for acknowledging the improvements incorporated into the revised manuscript. We have aimed to present the methodology and experimental evaluation more clearly and to provide a more explicit discussion of the study’s limitations and future research directions.

****

**Reviewer Concern # 9:** **The manuscript is logically organized, technically relevant, and contributes meaningfully to research on AI-assisted healthcare decision support.**

**Author response:** 

 We sincerely appreciate the reviewer’s positive evaluation of the manuscript’s organization, relevance, and contribution. We are pleased that the proposed HRAESN framework and its experimental evaluation are considered relevant to research on heart disease prediction.Strengthened novelty discussion and limitations.

****

**Reviewer Concern # 10:** **Overall, the article is suitable for indexing, and I recommend its acceptance for indexing as a valuable contribution to deep learning-based heart disease prediction research.**

**Author response:** 

 We thank the reviewer for the positive overall assessment of our manuscript and for the recommendation in its favour. We appreciate the reviewer’s recognition of the relevance of the proposed HRAESN framework, the experimental evaluation, and the contribution to heart disease prediction research.

****

# Reviewer report: Reviewer response for version 2

**DOI:** 10.5256/f1000research.187669.r414780

**Published:** 2025-09-25

**Authors:** Dhadkan Shrestha

**Affiliations:** 1 Texas State University College of Science and Engineering, San Marcos, Texas, USA

Everything looks good now. Thank you for revising.

Is the work clearly and accurately presented and does it cite the current literature?

Partly

If applicable, is the statistical analysis and its interpretation appropriate?

Partly

Are all the source data underlying the results available to ensure full reproducibility?

Yes

Is the study design appropriate and is the work technically sound?

Partly

Are the conclusions drawn adequately supported by the results?

Partly

Are sufficient details of methods and analysis provided to allow replication by others?

Partly

Reviewer Expertise:

Machine Learning, Artificial Intelligence, Big Data

I confirm that I have read this submission and believe that I have an appropriate level of expertise to confirm that it is of an acceptable scientific standard.

# Response

**Published:** 2025-09-25

**Authors:** VIJAYA ARJUNAN RANGANATHAN

**Affiliations:** School of Computer Engineering, Manipal Academy of Higher Education, Manipal, Karnataka, India

Thank you for your kind feedback and approval.

# Reviewer report: Reviewer response for version 1

**DOI:** 10.5256/f1000research.182263.r403945

**Published:** 2025-09-04

**Authors:** MUHAMMAD HAMMAD MEMON

**Affiliations:** 1 Southwest University of Science and Technology, Sichuan, China

**Summary of the Article:**

 The manuscript introduces a **Hybrid Residual Attention with Echo State Network (HRAESN)** model for ischemic heart disease (IHD) prediction. The model integrates **Attention Residual Learning (ARL)** to enhance feature extraction and **Echo State Networks (ESN)** for efficient time-series processing. Two datasets are used: the Kaggle Cardiovascular Disease dataset (70,000 samples) and the UCI Heart Disease dataset (303 samples). The authors report **very high performance** (up to 98.4% accuracy), claiming that HRAESN outperforms traditional ML/DL baselines.

 The study is relevant and well-motivated, with clear clinical importance. However, there are major concerns regarding **methodological rigor, reproducibility, and statistical robustness**.

**Major Concerns**

1. **Presentation and Literature Coverage**
  - The manuscript is generally clear, but the **literature review is overly descriptive** and includes some **weak references** (e.g., tutorial websites).
  - Prior work on combining attention and ESN (e.g., Deep Belief Echo-State Networks, Graph Residual Attention) is not sufficiently discussed. The novelty contribution must be **better distinguished**.
2. **Study Design and Technical Soundness**
  - The reported performance (>97% accuracy) is **unrealistically high** for these datasets and suggests possible **overfitting or data leakage**.
  - Only a single **80:20 train-test split** is reported. This is not sufficient for robust evaluation in medical ML. At minimum, **k-fold cross-validation** with stratified sampling is required.
3. **Methods and Replication**
  - Details of the **Ischemic Heart Disease Multiple Imputation Technique** are insufficient. The method is referenced but not described in reproducible detail.
  - No **code, model weights, or supplementary scripts** are provided, making replication difficult.
4. **Statistical Analysis**
  - No **statistical significance testing** (e.g., McNemar’s test, paired t-test, Wilcoxon signed-rank test) is provided. Reported differences may not be statistically meaningful.
  - Metrics such as **ROC curves, AUC, calibration plots, and precision-recall curves** should be included for clinical interpretability.
5. **Reproducibility and Source Data**
  - Although the datasets are public, the **exact preprocessing steps and imputation pipeline are not fully transparent**, which limits reproducibility.
  - PCA plots and confusion matrices are shown but lack supporting raw numbers or code availability.
6. **Support for Conclusions**
  - While results are promising, conclusions about clinical utility are **overstated**. Without independent external validation on real hospital datasets, it is premature to suggest readiness for clinical deployment.
  - Limitations such as **dataset imbalance, computational cost, and lack of external validation** are only briefly acknowledged and need stronger discussion.

**Minor Comments**

- Some sections could be streamlined (particularly Related Works).
- Figures would benefit from **statistical annotations** (e.g., significance levels).
- The **ethics statement** should clarify whether the Kaggle dataset contributor had appropriate institutional approval.
- Writing is generally clear but could be **more concise** in parts.

**Recommendations to Improve the Manuscript**

1. Re-run experiments with **10-fold cross-validation** and report mean ± standard deviation.
2. Add **statistical tests** to confirm whether improvements over baselines are significant.
3. Provide **algorithmic details** of the imputation method and release **source code/models**.
4. Include **AUC/ROC, calibration, and PR curves** for stronger evaluation.
5. Strengthen the **novelty discussion** by differentiating HRAESN from earlier ESN+attention studies.
6. Expand the **limitations section**, especially regarding generalizability and clinical applicability.

**Final Recommendation**

**Major Revision**

 The study addresses an important healthcare challenge and proposes an interesting hybrid deep learning approach. However, **methodological rigor, reproducibility, and statistical analysis must be improved** to make the findings scientifically sound and credible for indexing.

Is the work clearly and accurately presented and does it cite the current literature?

Partly

If applicable, is the statistical analysis and its interpretation appropriate?

Partly

Are all the source data underlying the results available to ensure full reproducibility?

Partly

Is the study design appropriate and is the work technically sound?

Partly

Are the conclusions drawn adequately supported by the results?

Partly

Are sufficient details of methods and analysis provided to allow replication by others?

Partly

Reviewer Expertise:

Artificial Intelligence and Machine Learning, Medical Data Mining and Predictive Analytics, Deep Learning for Healthcare Applications, Network Security and Cloud Computing.

I confirm that I have read this submission and believe that I have an appropriate level of expertise to state that I do not consider it to be of an acceptable scientific standard, for reasons outlined above.

# Response

**Published:** 2025-09-10

**Authors:** VIJAYA ARJUNAN RANGANATHAN

**Affiliations:** School of Computer Engineering, Manipal Academy of Higher Education, Manipal, Karnataka, India

**Deep Learning based hybrid residual attention and echo state network for high-accuracy heart disease prediction**

**Responses to peer review reports**

**Reviewer#3, Concern # 1:** **Summary of the Article:**

**Author response:**  We thank Reviewer  for the detailed assessment. The reviewer highlighted the clinical relevance of our work while raising concerns about methodology, reproducibility, and statistical robustness. We carefully revised the manuscript to address all points raised. Below we provide a structured response.

****

**Reviewer#3, Concern # 2:** **Presentation and Literature Coverage**

**Author response:**  We acknowledge this important observation. The Related Works section has been streamlined and focused on high-quality peer-reviewed studies. We expanded discussion of prior attention+ESN combinations, including Deep Belief Echo-State Networks (DBEN) and Graph Residual Attention models, to clearly distinguish our contribution. Our novelty lies in extending ESNs beyond time-series into structured clinical tabular data, integrated with ARL and combined with a tailored imputation framework.

**Author action: **Revised Section 2 (Related Works) to be more concise, replaced weak/tutorial references with peer-reviewed sources, and explicitly clarified novelty.

****

**Reviewer#3, Concern # 3:** **Study Design and Technical Soundness**

**Author response:**  We appreciate this concern. To strengthen robustness, we re-ran experiments with 5-fold and 10-fold stratified cross-validation in addition to the 80:20 split. Results are now reported as mean ± standard deviation. Performance remained consistently high, though slightly lower than single-split values, confirming stability without evidence of leakage.

**Author action: **Added cross-validation experiments and updated Tables 7–10 with mean ± SD. Reproducibility pipeline clarified in Section 3.5 Methodology.

****

**Reviewer#3, Concern # 4:** **Methods and Replication**

**Author response:**  We agree. The Ischemic Heart Disease Multiple Imputation Technique (IHD-MIT) is now described in step-by-step detail (predictor selection, iterative regression, variance preservation). For transparency, we have expanded the methodological description of the IHD-MIT imputation pipeline and model implementation in detail.

**Author action: **Expanded Section 3.5.1 (IHD-MIT) with algorithmic details.

****

**Reviewer#3, Concern # 5:** **Statistical Analysis**

**Author response:**  We fully agree. We added statistical significance testing (McNemar’s test for paired predictions, Wilcoxon signed-rank across folds) to confirm differences. Additionally, we now report ROC curves, AUC values, and calibration plots for clinical interpretability. Results demonstrate that HRAESN improvements are statistically significant (p < 0.05).

**Author action: **Added Figure 7 for ROC/AUC; included calibration analysis. Expanded Results Section 4.2–4.3 to include statistical testing.

****

**Reviewer#3, Concern # 6:** **Reproducibility and Source Data**

**Author response:**  We clarified all preprocessing steps, including normalization, imputation, train-test stratification, and cross-validation.

**Author action: **Updated Figures 3–6 captions with supporting details.

****

**Reviewer#3, Concern # 7:** **Support for Conclusions**

**Author response:**  We agree and have moderated claims. We now clearly state that this work is a proof-of-concept and not clinically deployable yet. We expanded Limitations to address external validation needs, potential bias from imputation, dataset imbalance, computational cost, and the need for interpretability studies.

**Author action: **Expanded Section 6 Limitations and Future Directions, emphasizing generalizability and next steps toward real-world validation.

****

**Reviewer#3, Concern # 8:** **Minor Comments**

**Author response:**  We thank the reviewer. Related Works was condensed (as above). Figures now include statistical annotations (significance levels). We clarified that Kaggle data are anonymized and released under open license, with ethical approvals obtained by original curators. The manuscript was carefully edited for conciseness.

**Author action: **Revised Section 2, updated figure annotations, clarified Ethics Statement, and streamlined prose throughout.

****

**Reviewer#3, Concern # 1:** **Reviewer Recommendations Implemented**

**Author response:** 

- Re-ran experiments with 10-fold cross-validation.
- Reported mean ± SD for all metrics.
- Added statistical tests (McNemar, Wilcoxon).
- Included ROC curves.
- Provided algorithmic details of IHD-MIT.
- Strengthened novelty discussion and limitations.

· ****

 We sincerely thank Reviewer  for the constructive and rigorous feedback. All major concerns regarding methodology, reproducibility, and statistical robustness have been addressed with substantial new analyses, expanded methodological detail, and clearer discussion of novelty and limitations. We believe these revisions significantly improve the scientific soundness and transparency of the manuscript.

# Reviewer report: Reviewer response for version 1

**DOI:** 10.5256/f1000research.182263.r406539

**Published:** 2025-09-04

**Authors:** Dhadkan Shrestha

**Affiliations:** 1 Texas State University College of Science and Engineering, San Marcos, Texas, USA

**1. Summary of the Article**

 The manuscript presents a **Hybrid Residual Attention with Echo State Network (HRAESN)** model for predicting ischemic heart disease (IHD). The approach integrates **Attention Residual Learning (ARL)** for feature extraction with **Echo State Networks (ESNs)** for efficient time-series learning. The study evaluates performance on two publicly available datasets: the **Kaggle Cardiovascular Disease dataset (70,000 records)** and the **UCI Heart Disease dataset (303 records)**. Missing values were handled using a tailored **Multiple Imputation Technique**. The proposed model achieved high classification performance, with accuracies of **98.4% (Kaggle)** and **97.7% (UCI)**, surpassing traditional ML and DL baselines. The authors conclude that the model offers strong potential as a clinical decision-support tool for early IHD detection.

**2. Evaluation of Key Criteria**

 (a) Clarity, Accuracy, and Literature Coverage

- **Assessment**: **Yes (with minor improvements suggested)**
  The manuscript is clearly written, structured logically, and cites a broad range of recent literature. The background is thorough and informative. A few parts (e.g., the objectives and problem statement) overlap slightly and could be streamlined for conciseness.
- **Constructive suggestions**:
  - Condense repetitive sections to make the narrative flow smoother.
  - More explicitly highlight how this approach differs from other recent hybrid deep learning works to strengthen the novelty claim.

(b) Study Design and Technical Soundness

- **Assessment**: **Yes**
  The study design is technically sound, and the proposed model is innovative. The integration of ARL and ESN is well motivated. The results are very strong, though the extremely high accuracy on the small UCI dataset raises the possibility of overfitting. Still, the use of dropout and a robust hyperparameter setup is a positive point.
- **Suggestions**:
  - For added robustness, apply **k-fold cross-validation** (especially for UCI dataset).
  - Briefly discuss class balance and whether any balancing strategy (e.g., weighting) was needed.

(c) Methods and Replicability

- **Assessment**: **Partly**
  The mathematical formulation is clear, and hyperparameters are well documented. This is very helpful. However, replication would be easier if code or pseudo-code for preprocessing and training were made available.
- **Suggestions**:
  - Consider providing **code, pseudocode, or a detailed pipeline** in supplementary materials.
  - Clarify how hyperparameters were tuned (manual search, grid search, etc.).

(d) Statistical Analysis and Interpretation

- **Assessment**: **Yes**
  The authors present a comprehensive set of performance metrics (accuracy, sensitivity, specificity, F1, Kappa, FAR/FRR), which is commendable. Interpretation is generally appropriate. One minor limitation is the absence of variance/confidence intervals across multiple runs.
- **Suggestions**:
  - Indicate whether results are from a single run or averaged across runs.
  - If possible, include confidence intervals or standard deviations.

(e) Availability of Source Data

- **Assessment**: **Yes**
  The datasets (UCI and Kaggle) are publicly available and properly cited. Ethical considerations are addressed. This ensures reproducibility of the raw data.
- **Suggestions**:
  - It would be helpful to share the **preprocessed datasets or preprocessing scripts** used before training.

(f) Conclusions and Support from Results

- **Assessment**: **Yes**
  The conclusions are well supported by the reported results. The performance improvement over baselines is clear. That said, claims about clinical applicability should be framed as **potential future applications** rather than immediate readiness.
- **Suggestions**:
  - Add a brief “Limitations” section noting that real-world hospital validation is pending.
  - Slightly temper statements on clinical deployment to emphasize this is a proof-of-concept

**3. Key Points to Address**

 To make the manuscript even stronger, the authors should consider:

1. Adding **cross-validation results** (especially for the UCI dataset).
2. Reporting **variance or confidence intervals** for performance metrics.
3. Providing **code/pseudocode or preprocessing details** for easier replication.
4. Including a short **limitations section** (dataset size, clinical validation, computational cost).

Is the work clearly and accurately presented and does it cite the current literature?

Partly

If applicable, is the statistical analysis and its interpretation appropriate?

Partly

Are all the source data underlying the results available to ensure full reproducibility?

Yes

Is the study design appropriate and is the work technically sound?

Partly

Are the conclusions drawn adequately supported by the results?

Partly

Are sufficient details of methods and analysis provided to allow replication by others?

Partly

Reviewer Expertise:

Machine Learning, Artificial Intelligence, Big Data

I confirm that I have read this submission and believe that I have an appropriate level of expertise to confirm that it is of an acceptable scientific standard, however I have significant reservations, as outlined above.

# Response

**Published:** 2025-09-10

**Authors:** VIJAYA ARJUNAN RANGANATHAN

**Affiliations:** School of Computer Engineering, Manipal Academy of Higher Education, Manipal, Karnataka, India

**Deep Learning based hybrid residual attention and echo state network for high-accuracy heart disease prediction**

**Responses to peer review reports**

**Reviewer#2, Concern # 1:** Summary of the Article

**Author response:**  We thank the reviewer for the accurate and concise summary of our work. We appreciate the recognition of our proposed Hybrid Residual Attention with Echo State Network (HRAESN) model, our methodological contributions (Attention Residual Learning combined with Echo State Networks), and our evaluation using the Kaggle and UCI datasets. We also thank the reviewer for noting our strategy for handling missing values and the strong performance achieved by the model.

****

**Reviewer#2, Concern # 2:** Clarity, Accuracy, and Literature Coverage

**Author response:**  We agree with this suggestion. We revised the Introduction to remove overlap between the problem statement and objectives, improving narrative flow. Additionally, we added a new paragraph at the end of the Introduction to explicitly highlight novelty: (i) integration of ARL with ESNs, (ii) extending ESNs to structured/tabular clinical data, and (iii) introducing an IHD-specific multiple imputation method.

**Author action: **Revised Introduction: merged problem statement + objectives into a concise paragraph. Added final paragraph in Introduction to emphasize novelty.

****

**Reviewer#2, Concern # 3:** Study Design and Technical Soundness

**Author response:**  We thank the reviewer for this important point. To address robustness, we added text in Methods clarifying that k-fold cross-validation (k=5) was performed on the UCI dataset, confirming stable results across folds. We also report class balance: UCI dataset (~54% IHD, ~46% healthy) and Kaggle dataset (~50% each), showing no major imbalance. No resampling or weighting was needed. We further acknowledge the potential risk of overfitting in the Discussion as a limitation.

**Author action: **Added in Section 3.5: description of k-fold cross-validation on UCI dataset. Added in Section 3.1.4: class balance description. Expanded Discussion: limitation noting overfitting risk in small datasets.

****

**Reviewer#2, Concern # 4:** Methods and Replicability

**Author response:**  We appreciate this suggestion. To enhance replicability, we included a pseudo-code style Algorithm (Algorithm 1) in the Methods section, summarizing the preprocessing, model training, and evaluation pipeline. We also clarified that hyperparameters were tuned via grid search, selecting the configuration with the highest validation F1-score.

**Author action: **Added Algorithm 1 (pipeline) in Section 3.5. Clarified hyperparameter tuning strategy (grid search).

****

**Reviewer#2, Concern # 5:** Statistical Analysis and Interpretation

**Author response:**  We agree. Results now explicitly state they are averaged across multiple runs. We also computed 95% confidence intervals for all primary metrics using bootstrap resampling (1000 iterations).

**Author action: **Updated Results to note averaged results across runs.

****

**Reviewer#2, Concern # 6:** Availability of Source Data

**Author response:**  We thank the reviewer for this comment. While raw datasets are already public, we recognize that preprocessing adds value for replication. We now provide a detailed preprocessing description in Methods (Section 3.1.4) and make scripts available upon request.

**Author action: **Expanded Section 3.1.4 with detailed preprocessing description.

****

**Reviewer#2, Concern # 7:** Conclusions and Support from Results

**Author response:**  We agree with this suggestion. The Discussion has been expanded with a new Limitations subsection addressing dataset size, lack of external clinical validation, potential imputation bias, and computational cost. Statements on clinical application have been revised to emphasize that this is a proof-of-concept with potential future clinical use.

**Author action: **Expanded Discussion with Limitations subsection. Rephrased Conclusion to emphasize proof-of-concept, not immediate deployment.

****

**Reviewer#2, Concern # 8:** Key Points to Address

**Author response:**  All these points have been addressed in the revision:

- Cross-validation results for UCI dataset included.
- 95% confidence intervals.
- Algorithm 1 (pseudo-code pipeline) added in Methods.
- Expanded Discussion with a Limitations section.

**Author action: **Revisions made in Sections 3.1.4, 3.5, Results, and Discussion.

****

 We sincerely thank Reviewer  for the constructive and rigorous feedback. All major concerns regarding methodology, reproducibility, and statistical robustness have been addressed with substantial new analyses, expanded methodological detail, and clearer discussion of novelty and limitations. We believe these revisions significantly improve the scientific soundness and transparency of the manuscript.

# Reviewer report: Reviewer response for version 1

**DOI:** 10.5256/f1000research.182263.r401540

**Published:** 2025-08-25

**Authors:** Amalie Dahl Haue

**Affiliations:** 1 University of Copenhagen, Copenhagen, Denmark

The research article by Ranganathan et al. presents a deep learning based hybrid residual attention and echo state network for high-accuracy heart disease prediction derived from analysis of the Kaggle Cardiovascular Disease dataset and the UCI Heart Disease dataset. Their model (HRAEN) demonstrates superior perfomance with accuracy rating between 97.7% and 98.4%.

 Introduction

 The very first paragraph is could benefit from being rewritten to ensure a better flow and updated to align with current practise. For example, neither stress test, nor Holter monitoring are used routinely to detect ischemic heart disease (IHD). Rather cardiac CT, RbPET and invasive examinations such as coronary arteriography are being used to assess degree of IHD

 Related works

 This section would benefit greatly from a more condensed presentation of the literature.

 Materials and methods

 It is not clear how heart disease (presence or absence) was defined in the two cohorts, i.e. which diagnostic tests were used.

 Figure 1 and 2 are not detailed enough, i.e. were all entries (observations) in the two datasets included in the study, what was the degree of missingness, and (again) how was IHD assessed?

 It is not clear how the Echo State Networkds (ESNs) were applied to the data at hand since not time-series data is introduced.

 Results and analysis

 The different classes are not annotated consistently. That is, is "Class 1" "heart disease" (as listed in Materials and methods) or "ischemic heart disease" (as listed in Results and analysis)?

 New metrics, such as Kappa score/coefficient and Jaccard coefficient are introduced in this section. They ought to be introduced in Materials and methods. 

 Figure 4: Does the figure display the performance of the models on a particular dataset or a combined version?

 For the performance metrics, it would be beneficial to include confidence intervals for assessment of statistical significance.

 Figure 6: The authors state that it converts that the proposed HRAESN model outperforms traditional classifiers in multiple performance aspects. However, only the HRAESN evaluated on the UCI and Kaggle dataset are reported in this figure.

 Were the test and training sets similar? It would be nice with a table that provides an overview of the baseline characteristics in the different populations.

 Table 6 and 7: The authors ought to argue that the HRAESN is comparable to the existing methods. For example, it is not clear why HRAESN on the UCI Heart Disease Dataset and the Kaggle Cardiovascular Disease dataset are being compared to different existing methods. Further, were the existing methods trained to perform a similar classification task as HRAESN. Again the definition of heart disease/IHD as how it was diagnosed is crucial here, but unfortunately lacking from this version of the manuscript.

 Discussion

 This section appears to be incomplete. For example, the lack of external validation is not addressed. Further, the use of imputation and the potential bias of the results is not discussed. Finally, there is not evaluation of the impact of the Attention Residual Learning (ARL), i.e., which features were most important in the classification when ARL was performed? And, could this strategy be used to identify a limited set of features that could obtain similar performance metrics?

Is the work clearly and accurately presented and does it cite the current literature?

Yes

If applicable, is the statistical analysis and its interpretation appropriate?

I cannot comment. A qualified statistician is required.

Are all the source data underlying the results available to ensure full reproducibility?

Yes

Is the study design appropriate and is the work technically sound?

Partly

Are the conclusions drawn adequately supported by the results?

Yes

Are sufficient details of methods and analysis provided to allow replication by others?

Partly

Reviewer Expertise:

Cardiology resident

I confirm that I have read this submission and believe that I have an appropriate level of expertise to confirm that it is of an acceptable scientific standard, however I have significant reservations, as outlined above.

# Response

**Published:** 2025-09-10

**Authors:** VIJAYA ARJUNAN RANGANATHAN

**Affiliations:** School of Computer Engineering, Manipal Academy of Higher Education, Manipal, Karnataka, India

**Deep Learning based hybrid residual attention and echo state network for high-accuracy heart disease prediction**

**Responses to peer review reports**

**Reviewer#1, Concern # 1:** Introduction

 The very first paragraph is could benefit from being rewritten to ensure a better flow and updated to align with current practise. For example, neither stress test, nor Holter monitoring are used routinely to detect ischemic heart disease (IHD). Rather cardiac CT, RbPET and invasive examinations such as coronary arteriography are being used to assess degree of IHD

**Author response:**  We thank the reviewer for this valuable suggestion. We have revised the introductory paragraph to better reflect current clinical practices, replacing outdated references (stress test, Holter monitoring) with contemporary modalities such as cardiac CT, RbPET, and coronary angiography.

**Author action: **The Introduction now begins with a discussion of ischemic heart disease pathophysiology and updated diagnostic modalities.

****

**Reviewer#1, Concern # 2: Related works**

**This section would benefit greatly from a more condensed presentation of the literature.**

**Author response:**  We appreciate this suggestion. We revised the Related Works section to streamline the narrative, grouping studies under thematic categories (traditional ML, deep learning, hybrid models, ESN-based, and attention-based methods).

**Author action: **Section 2 was restructured for conciseness while retaining comprehensiveness.

****

**Reviewer#1, Concern # 3:** Materials and methods

 It is not clear how heart disease (presence or absence) was defined in the two cohorts, i.e. which diagnostic tests were used.

**Author response:**  We acknowledge this concern. We now clearly define the target variables in both datasets:

- UCI: angiography-based “num” variable, binarized (0 = absence, 1–4 = presence of disease).
- Kaggle: “cardio” variable defined by combined clinical assessments (blood pressure, cholesterol, ECG).

**Author action: **Added Section 3.1.4 Definition of Heart Disease in the Datasets.

****

**Reviewer#1, Concern # 4: **Figure 1 and 2 are not detailed enough, i.e. were all entries (observations) in the two datasets included in the study, what was the degree of missingness, and (again) how was IHD assessed?

**Author response:**  We appreciate this important comment. We have clarified in Section 3.1.4 how IHD was defined in each dataset (Kaggle: cardio; UCI: num attribute binarized). Missing data handling using the IHD Multiple Imputation Technique is now described. We also added clarification on how Echo State Networks were applied to structured tabular data (not time-series). Figures 1 and 2 were redesigned to show dataset composition, preprocessing, and architecture in greater detail.

**Author action: **Section 3.1.4 updated with disease definition, missingness handling, and ESN applicability.

 Redesigned Figure 1 (workflow with dataset size, missing values, preprocessing, labels, metrics).

 Redesigned Figure 2 (detailed HRAESN architecture with ESN + ARL modules).

****

**Reviewer#1, Concern # 5:** It is not clear how the Echo State Networkds (ESNs) were applied to the data at hand since not time-series data is introduced.

**Author response:**  We agree this required clarification. While raw ECG series were not used, we adapted ESNs by treating patient feature vectors as structured sequences, mapping them into reservoir states to capture nonlinear feature dependencies.

**Author action: **Added explanation in Section 3.5 Methodology – Application of ESNs to Tabular Data.

****

**Reviewer#1, Concern # 6:** Results and analysis

 The different classes are not annotated consistently. That is, is "Class 1" "heart disease" (as listed in Materials and methods) or "ischemic heart disease" (as listed in Results and analysis)?

**Author response:**  We standardized terminology throughout: Class 0 = no IHD, Class 1 = IHD present.

**Author action: **Updated class definitions consistently across Materials & Methods, Results, and figures.

****

**Reviewer#1, Concern # 7:** New metrics, such as Kappa score/coefficient and Jaccard coefficient are introduced in this section. They ought to be introduced in Materials and methods. 

**Author response:**  We thank the reviewer. These metrics are now introduced in Evaluation Metrics subsection of Materials and Methods.

**Author action: **Section 3.5 includes definitions of Kappa coefficient and Jaccard index.



**Reviewer#1, Concern # 8:** Figure 4: Does the figure display the performance of the models on a particular dataset or a combined version?

**Author response:**  We have clarified the figure captions to indicate that Figure 4 reports performance metrics separately for both UCI and Kaggle datasets.

**Author action: **Updated Figure 4 caption as suggested

****

**Reviewer#1, Concern # 9:** For the performance metrics, it would be beneficial to include confidence intervals for assessment of statistical significance.

**Author response:**  We have now reported 95% confidence intervals using bootstrap resampling (1000 iterations) for all major performance metrics.

**Author action: **Confidence intervals are included in tables as suggested.

****

**Reviewer#1, Concern # 10:** Figure 6: The authors state that it converts that the proposed HRAESN model outperforms traditional classifiers in multiple performance aspects. However, only the HRAESN evaluated on the UCI and Kaggle dataset are reported in this figure.

**Author response:**  We agree this was ambiguous. Figure 6 is intended to illustrate HRAESN error rates across datasets, while comparative results with baselines are in Tables 8–9.

**Author action: **Figure 6 caption updated to clarify scope

****

**Reviewer#1, Concern # 11:** Were the test and training sets similar? It would be nice with a table that provides an overview of the baseline characteristics in the different populations.

**Author response:**  We now provide a table of baseline characteristics (age, sex, cholesterol, blood pressure) for training and test subsets.

**Author action: **Added Table 3: Baseline Characteristics.



**Reviewer#1, Concern # 12:** Table 6 and 7: The authors ought to argue that the HRAESN is comparable to the existing methods. For example, it is not clear why HRAESN on the UCI Heart Disease Dataset and the Kaggle Cardiovascular Disease dataset are being compared to different existing methods. Further, were the existing methods trained to perform a similar classification task as HRAESN. Again the definition of heart disease/IHD as how it was diagnosed is crucial here, but unfortunately lacking from this version of the manuscript.

**Author response:** We appreciate this comment. Tables 6 and 7 were updated/clarified with consistent captions and explanations of dataset comparisons.  We expanded the Discussion to address:

- Lack of external validation and need for future hospital-based datasets.
- Potential imputation bias and future use of sensitivity analyses.
- Importance of ARL interpretability and plans to evaluate feature contributions.

**Author action: **Tables 6–7 revised with rationale for comparing UCI vs Kaggle against different baselines. Expanded Section 6 Limitations and Future Directions.



**Reviewer#1, Concern # 13:** Discussion

 This section appears to be incomplete. For example, the lack of external validation is not addressed. Further, the use of imputation and the potential bias of the results is not discussed. Finally, there is not evaluation of the impact of the Attention Residual Learning (ARL), i.e., which features were most important in the classification when ARL was performed? And, could this strategy be used to identify a limited set of features that could obtain similar performance metrics?

**Author response:**  We agree with this comment and have expanded the Discussion to address these limitations. We now discuss the absence of external validation, the potential bias introduced by imputation, and the interpretability of ARL. We also comment on future work to explore feature importance and whether a smaller subset of features could achieve comparable accuracy.

**Author action: **Discussion section expanded with subsections on limitations, imputation bias, and ARL interpretability.



 We sincerely thank Reviewer  for the constructive and rigorous feedback. All major concerns regarding methodology, reproducibility, and statistical robustness have been addressed with substantial new analyses, expanded methodological detail, and clearer discussion of novelty and limitations. We believe these revisions significantly improve the scientific soundness and transparency of the manuscript.
